# A framework for estimating the US mortality burden of fine particulate matter exposure attributable to indoor and outdoor microenvironments

**DOI:** 10.1038/s41370-018-0103-4

**Published:** 2018-12-05

**Authors:** Parham Azimi, Brent Stephens

**Affiliations:** Department of Civil, Architectural, and Environmental Engineering, Illinois Institute of Technology, Chicago, IL USA

**Keywords:** criteria pollutants, epidemiology, exposure modeling, inhalation exposure, particulate matter

## Abstract

Exposure to fine particulate matter (PM_2.5_) is associated with increased mortality. Although epidemiology studies typically use outdoor PM_2.5_ concentrations as surrogates for exposure, the majority of PM_2.5_ exposure in the US occurs in microenvironments other than outdoors. We develop a framework for estimating the total US mortality burden attributable to exposure to PM_2.5_ of both indoor and outdoor origin in the primary non-smoking microenvironments in which people spend most of their time. The framework utilizes an exposure-response function combined with adjusted mortality effect estimates that account for underlying exposures to PM_2.5_ of outdoor origin that likely occurred in the original epidemiology populations from which effect estimates are derived. We demonstrate the framework using several different scenarios to estimate the potential magnitude and bounds of the US mortality burden attributable to total PM_2.5_ exposure across all non-smoking environments under a variety of assumptions. Our best estimates of the US mortality burden associated with total PM_2.5_ exposure in the year 2012 range from ~230,000 to ~300,000 deaths. Indoor exposure to PM_2.5_ of outdoor origin is typically the largest total exposure, accounting for ~40–60% of total mortality, followed by residential exposure to indoor PM_2.5_ sources, which also drives the majority of variability in each scenario.

## Introduction

Elevated outdoor concentrations of fine particulate matter (i.e., the mass concentration of particles ≤ 2.5 µm in aerodynamic diameter; PM_2.5_) have been consistently associated with increased mortality in numerous epidemiology studies [1–9]. Although epidemiology studies typically use centrally monitored outdoor PM_2.5_ concentrations as surrogates for average human exposures to PM_2.5_ of outdoor origin, the majority of exposure to PM_2.5_ of outdoor origin in the US and other industrialized nations typically occurs in various other microenvironments, including inside residences, offices, schools, and vehicles [10–16]. This is because people spend the majority of their time in microenvironments other than outdoors [17, 18] and outdoor PM_2.5_ can infiltrate and persist into different microenvironments with varying efficiencies [19–24]. There are also many PM_2.5_ sources present in non-smoking indoor microenvironments, including cooking [25–27], burning incense and candles [28, 29], operating office equipment [30, 31], resuspension from settled dust from human activities such as walking and cleaning [32, 33], and secondary organic aerosols from oxidation reactions [34]. To date, the vast majority of air pollution epidemiology studies and quantitative risk assessments have not explicitly accounted for these varied microenvironmental exposures [35, 36].

The objective of this work is to develop a framework for estimating the total US mortality burden attributable to exposure to PM_2.5_ of both indoor and outdoor origin in the primary non-smoking microenvironments in which people spend most of their time. The framework primarily utilizes a modified version of an exposure-response function commonly used for air pollution risk assessment combined with adjusted mortality effect estimates that account for estimates of underlying microenvironmental exposures to PM_2.5_ of outdoor origin that likely occurred in prior epidemiology cohort studies. We demonstrate the utility of the framework by conducting several scenario analyses to estimate the likely magnitude and bounds of the US mortality burden associated with long-term PM_2.5_ exposures that result from both indoor and outdoor PM sources in each microenvironment. While no single model scenario is considered to be the definitive representation of the US mortality burden of microenvironmental PM_2.5_ exposures due to unique data limitations in each case, each model scenario offers insight into how the framework can be used with richer data sets in the future to refine nationwide mortality estimates and ultimately to inform policy decisions to reduce exposures in the microenvironments in which they most often occur.

## Materials and methods

### Selection of an appropriate exposure-response function

Integral to the model framework is the selection of an appropriate health impact function. A number of recent air pollution risk assessments have estimated mortality and/or morbidity associated with ambient PM_2.5_ exposure in various locations using different forms of health impact functions and associated effect estimates derived from epidemiology studies. Historically, most studies have used a variant of a generic exposure-response health impact function for ambient air pollution [37] to estimate a population’s change in health endpoint (Δ*y*_*i*_) due to a change in the assumed population-average exposure to pollutant *i* (Δ*E*_*i*_) (e.g., Eq. 1).

1 $$\Delta y_i = y_0\left[ {{\mathrm{exp}}\left( {\beta _i \times \Delta E_i} \right) - 1} \right]Pop$$

where *y*_0_ is the annual baseline prevalence of illness (per person per year), *β*_*i*_ is the health endpoint effect estimate for pollutant *i* resulting from prior epidemiology studies (e.g., per μg/m^3^ of pollutant *i*), Δ*E*_*i*_ is the change in exposure concentration relative to an assumed baseline or threshold concentration (e.g., μg/m^3^ of pollutant *i*, typically assuming outdoor concentrations are surrogates for exposure), and *Pop* is size of the affected population. This approach has been used recently to estimate the mortality burden associated with outdoor PM_2.5_ concentrations in the US [38–42] and globally [43, 44]. For example, Fann et al. (2017) [42] used this approach with all-cause mortality effect estimates from Krewski et al. (2009) [5] to estimate that ~120,000 deaths (95% CI: 83,000–160,000) were associated with outdoor PM_2.5_ exposures in the in 2010. Fann et al. (2017) [42] also made another estimate of ~200,000 (95% CI: 43,000–1,100,000) deaths associated with outdoor PM_2.5_ using a different model form and effect estimates from Nasari et al. (2016) [45]. Similar approaches have also recently been extended to estimate the chronic health burden associated with long-term indoor PM exposures using effect estimates taken directly from the outdoor air epidemiology literature [46–50].

Another widely used approach to air pollution risk assessment is the Global Burden of Disease (GBD) study’s integrated exposure-response (IER) methodology [51–56], and its follow-up Global Exposure Mortality Model (GEMM) [57], which were developed in part because the generic expression in Eq. 1 is based on epidemiology cohort studies in the US and Europe with outdoor PM_2.5_ concentrations (typically below 30 µg/m^3^) that may not be representative for countries with much higher ambient air pollution levels [53] or for other, higher, PM_2.5_ exposures such as secondhand or active smoking. Here we primarily utilize a modified version of the generic exposure-response health impact function in Eq. 1 for the model framework because (a) it was developed for use with epidemiology studies with PM_2.5_ concentrations within the range of concern in non-smoking indoor and outdoor microenvironments in the US, (b) there is considerable uncertainty in the shape of the GBD IER function and its fitted parameters at lower PM_2.5_ concentrations most relevant to this study, and (c) it has been used successfully in other recent indoor microenvironmental exposure investigations. However, we also apply the IER model and evaluate its utility in the SI.

### Modifying the exposure-response function

We modify the exposure-response function in Eq. 1 for PM_2.5_ in a manner similar to that in Logue et al. (2012) [48] to account for microenvironmental PM_2.5_ concentrations and exposures, albeit with a few additional modifications as shown in Eq. 2. First, we introduce a modified form of *β*_*i*_ for ambient-generated PM_2.5_ (i.e., *β*_*PM2.5,AG,modified*_) to account for estimates of the underlying long-term average exposures to PM_2.5_ of outdoor origin that likely occurred in various microenvironments in the cohort populations used in the original epidemiology studies from which *β*_*PM2.5*_ was derived. This modification provides an adjusted effect estimate for outdoor PM_2.5_ based on estimates of long-term average microenvironmental exposures that can be more universally applied to other microenvironmental exposure estimates rather than using outdoor PM_2.5_ concentrations alone as a surrogate for exposure.

Second, we separately account for long-term average PM_2.5_ exposures above an assumed threshold concentration in each microenvironment *j* that result from ambient-generated sources (Δ*C*_*PM2.5,AG,j*_) and indoor-generated sources (Δ*C*_*PM2.5,IG,j*_). Third, *t*_*j*_ accounts for the average fraction of time spent in a particular microenvironment *j*. Thus, the sums of Δ*C*_*PM2.5,AG,j*_ *×* *t*_*j*_ and Δ*C*_*PM2.5,IG,j*_ *×* *t*_*j*_ across all microenvironments more realistically account for total PM_2.5_ exposure (Δ*E*_*PM2.5*_) from both indoor and outdoor sources. Finally, we also allow for using different assumptions for modified mortality effect estimates for ambient-generated and indoor-generated PM_2.5_ (i.e., *β*_*PM2.5,AG,modified*_ and *β*_*PM2.5,IG,modified*_, respectively). Although the framework can account for varying toxicity of ambient- and indoor-generated PM_2.5_, we assume equal toxicity here because of conflicting conclusions among the limited number of studies that have investigated differential toxicity using paired indoor, outdoor, and/or personal PM samples [58–63].

2 $$\Delta y_{PM2.5} = y_0\left[ \exp \left( \beta _{PM2.5,IG,modified} {\!} \times {\!} \mathop {\sum }\limits_j (\Delta C_{PM2.5,IG,j} {\!} \times {\!} t_j) \right.\right. \\ + \left. \left. \beta _{PM2.5,AG,modified} \times \mathop {\sum }\limits_j (\Delta C_{PM2.5,AG,j} \times t_j) \right) - 1 \right]Pop$$

We consider four main microenvironments in which people are exposed to PM_2.5_ of both indoor and outdoor origin: (i) inside residences, (ii) inside indoor environments other than residences (e.g., schools, business, restaurants, etc.), (iii) inside vehicles, and (iv) outdoors. Equation 3 shows modified forms of the Σ(Δ*C*_*PM2.5,IG,j*_*×t*_*j*_) and Σ(Δ*C*_*PM2.5,AG,j*_*×t*_*j*_) terms in Eq. 2 that account for the long-term average PM_2.5_ concentrations resulting from both indoor and outdoor sources and the average fraction of time spent inside each of these four primary microenvironments.

3a $$\begin{array}{l}\mathop {\sum }\limits_j (\Delta C_{PM2.5,IG,j} \times t_j) = \left( {\Delta C_{PM2.5,IG,residences} \times t_{residences}} \right)\\ + \left( {\Delta C_{PM2.5,IG,other\,indoor} \times t_{other\,indoor}} \right)\end{array}$$

3b $$\begin{array}{l}\mathop {\sum }\limits_j (\Delta C_{PM2.5,AG,j} \times t_j) = \left( {\Delta C_{PM2.5,AG,residences} \times t_{residences}} \right) \\ + \left( {\Delta C_{PM2.5,AG,other\,indoor} \times t_{other\,indoor}} \right)\\ + \left( {\Delta C_{PM2.5,AG,vehicles} \times t_{vehicles}} \right) + (\Delta C_{PM2.5,outdoor} {\!} \times {\!} t_{outdoor})\end{array}$$

where Δ*C*_*PM2.5,IG,residences*_ and Δ*C*_*PM2.5,IG,other indoor*_ are the differences in long-term average concentrations of indoor-generated PM_2.5_ in non-smoking residences and all other non-smoking indoor environments other than residences, respectively, both compared to a baseline value in which there are no indoor PM_2.5_ sources (μg/m^3^); Δ*C*_*PM2.5,AG,residences*_, Δ*C*_*PM2.5,AG,other indoor*_, and Δ*C*_*PM2.5,AG,vehicles*_ are the differences in long-term average concentrations of ambient-generated PM_2.5_ in residences, indoor environments other than residences, and vehicles, respectively, compared to a baseline value (μg/m^3^); Δ*C*_*PM2.5,outdoor*_ is the difference in long-term average outdoor PM_2.5_ concentrations also compared to a baseline value (μg/m^3^); and *t*_*residences*_, *t*_*other indoor*_, *t*_*vehicles*_, and *t*_*outdoor*_ are the long-term average fractions of time spent inside each microenvironment, respectively. Note that Eq. 3a assumes there are no indoor sources of PM_2.5_ inside vehicles, primarily because of a lack of comprehensive surveys of in-vehicle PM sources, although several studies have shown that in-vehicle PM_2.5_ exposures can be higher than the near-roadway exposures in some circumstances [64, 65].

### Modifying effect estimates for PM_2.5_ of outdoor origin

Data from the 1992–1994 National Human Activity Pattern Survey (NHAPS) showed that, on average, people in the US spent 68.7% of their time in residences, 18.2% of their time in indoor locations other than residences (e.g., offices, factories, bars, schools, and restaurants), 5.5% of their time in vehicles, and 7.6% of their time outdoors [17]. Therefore, historically observed associations between outdoor PM_2.5_ concentrations and adverse health outcomes can reasonably be expected to have indirectly accounted for the underlying exposures to PM_2.5_ of outdoor origin that infiltrates and persists in these various microenvironments [66]. Failing to account for these underlying exposures to PM_2.5_ of outdoor origin in different microenvironments can lead to exposure misclassification and errors in effect estimates [35, 67–80]. To account for this phenomenon, we developed a modified mortality effect estimate for PM_2.5_ of outdoor origin (i.e., *β*_*PM2.5,AG,modified*_) based on the average fraction of PM_2.5_ of outdoor origin that infiltrates and persists in each assumed microenvironment (i.e., the infiltration factor) combined with the average fraction of time spent in each microenvironment, as shown in Eq. 4.

4 $$\beta _{PM2.5,AG,modified} = \frac{{\beta _{PM2.5}}}{{{\mathrm{\Sigma }}F_jt_j}}$$

where *β*_*PM*2.5_ is the mortality effect estimate for outdoor PM_2.5_ from epidemiology studies that used outdoor concentrations as surrogates for average population exposure to outdoor PM_2.5_, *F*_*j*_ is the average PM_2.5_ infiltration factor for microenvironment *j*, and *t*_*j*_ is the fraction of time spent in each microenvironment *j*. Σ*F*_*j*_×*t*_*j*_ is estimated using Eq. 5, which represents a weighted average of the product of the infiltration factors and fractional time spent in each of the four microenvironments used herein.

5 $${\mathrm{\Sigma }}F_j \times t_j = (F \times t)_{outdoor} + (F \times t)_{residence} + (F \times t)_{vehicle} \\ + (F \times t)_{other\,indoor}$$

We estimate a mean value of Σ*F*_*j*_×*t*_*j*_ to be ~0.60 for the US population using a number of data sources as described in the SI. Although there would be variability in this value for each individual in a particular population included in a cohort study, this value is assumed to be broadly applicable as a reasonable estimate of the population-average value.

### Applying the model framework: scenario analyses

We apply the model framework using MATLAB to estimate the magnitude and bounds of the US mortality burden of long-term average total PM_2.5_ exposures that result from indoor and outdoor PM sources in all non-smoking microenvironments. We define two primary scenarios that involve different assumptions and data sources for key input parameters, including: (i) a nationwide estimate based primarily on data from field measurements (where possible) and nationwide distributions of model input parameters; and (ii) a nationwide estimate based primarily on regionally varying modeled microenvironmental PM_2.5_ concentrations and other regionally varying model input parameters (where possible). A third scenario involves an application of the GBD IER model for comparison purposes; methods and results are included in the SI (although we have limited confidence in the approach for a number of reasons as described in the SI). Each model scenario is constructed to yield insight into how the framework can be used to generate mortality estimates and attribute them to microenvironmental exposures, while also highlighting unique data limitations present within each set of scenario assumptions.

For both Scenario 1 and 2, we use a central pooled estimate of RR for the increase in long-term all-cause mortality associated with outdoor PM_2.5_ concentrations in the US of 7.3% per 10 µg/m^3^ (95% CI: 3.7–11%) as reported in a recent quantitative meta-analysis of outdoor PM_2.5_ C-R functions [39]. We convert the pooled RR estimate of 1.073 per 10 µg/m^3^ to an effect estimate (i.e., *β*_*PM2.5*_) of 0.0070 (95% CI: 0.0036–0.0104), where *β*_*PM2.5*_ *=* ln(RR)/10 [81]. We fit a Weibull distribution to these reported values, resulting in a mean (±SD) value of *β*_*PM2.5*_ = 0.0070 (± 0.0016) per µg/m^3^ with distribution shape factors of *α* = 0.765 and *β* = 4.95. A Weibull distribution was used because it yields a distribution that is very close to normal in shape, but does not produce any negative values. Moreover, we estimate *β*_*PM2.5,AG,modified*_ to be ~0.0117 per µg/m^3^ using Eq. 4 (i.e., 0.0070 divided by 0.6) with a 95% CI of 0.0060–0.0174 per µg/m^3^. This modified effect estimate for all-cause mortality associated with outdoor PM_2.5_ represents a more generalizable effect estimate that accounts for the population-average locations and durations in which people are likely exposed to PM_2.5_ of outdoor origin.

#### Scenario 1: Nationwide estimate based primarily on prior field studies

In Scenario 1, we estimated the mortality burden for the adult population 35 years and older using nationwide distributions of model inputs. We assumed a national annual average outdoor PM_2.5_ concentration of 9.1 µg/m^3^ with 10th and 90th percentiles of 6.6 and 11.2 µg/m^3^, respectively, taken from the EPA’s nationwide monitoring network data for the year 2012 [82]. The year 2012 was chosen because it was the year for which we had the most comprehensive national (Scenario 1) and regional (Scenario 2) estimates for indoor and outdoor PM_2.5_ concentrations. We fit a lognormal distribution through the reported arithmetic mean and percentiles to construct a distribution from which to sample (GM = 8.84 µg/m^3^ and GSD = 1.246). We assumed a baseline (i.e., threshold) PM_2.5_ concentration of zero in each microenvironment, which is consistent with other recent applications of the core health impact function used in this scenario [42, 43] and with a number of studies that suggest there is no evidence of a population threshold in the relationship between long-term exposure to ambient PM_2.5_ and mortality [83–86]. We assumed that the 2012 nationwide population (*Pop*) and mortality rate (*y*_0_) for persons 35 years and older were 166,516,716 and 1.463 × 10^−2^ per person per year, respectively, using data from the CDC WONDER system [87].

We used Monte Carlo simulations with 10,000 iterations to sample from what we assumed for the purposes of Scenario 1 to be nationally representative distributions of every other model input parameter, including modified PM_2.5_ mortality effect estimates (described previously), time-activity patterns, and estimates of long-term average PM_2.5_ concentrations of both indoor and outdoor origin in each microenvironment taken largely from prior field measurements. There are three versions of Scenario 1, each of which involved sampling from different distributions to estimate residential PM_2.5_ concentrations of both indoor and outdoor origin. We sampled data from (i) the Relationship of Indoor, Outdoor and Personal Air (RIOPA) [13] and (ii) the Multi-Ethnic Study of the Atherosclerosis and Air Pollution (MESA Air) [18, 19] studies independently, as well as (iii) both RIOPA and MESA equally. Briefly, the RIOPA study measured indoor and outdoor PM_2.5_ concentrations concurrently for 48 h in 212 non-smoking residences in three US cities, while MESA Air measured indoor and outdoor PM_2.5_ concentrations concurrently over a 2-week period in 208 homes in warm seasons and 264 homes in cold seasons in seven US cities. Crucially, subsequent analyses of both data sets reported distributions of PM_2.5_ infiltration factors, which can be used to estimate the relative contributions of both indoor and outdoor sources to indoor PM_2.5_ concentrations in the sample residences. Although a few other studies have also explicitly measured indoor concentrations of PM_2.5_ in US residences resulting from indoor and outdoor sources, including a study of 294 inner-city homes of children with asthma in seven cities [27] and 68 smoking and non-smoking homes in six cities [88], we chose to rely on the RIOPA and MESA Air studies because they included large sample sizes of non-smoking homes occupied by adults in multiple US cities.

All relevant model inputs and data sources for Scenario 1 are summarized in full in the SI. Each model iteration represents a population-level estimate of total mortality summed across all microenvironmental exposures; thus, the central tendency of the model output provides the most likely estimate of the magnitude of the total mortality associated with PM_2.5_ exposure and the output range informs the likely bounds of that estimate. In all microenvironments, if a sampled value of a microenvironmental PM_2.5_ concentration was a negative value, it was replaced with zero.

#### Scenario 2: Nationwide estimate based on regional model outputs

In Scenario 2, we similarly applied the model framework to make a nationwide estimate of the total mortality burden attributable to microenvironmental PM_2.5_ exposures, albeit using regional assumptions for some input parameters for which regional data were available, including population demographics, baseline over-35 adult mortality rates, outdoor PM_2.5_ concentrations, and, importantly, residential indoor PM_2.5_ concentrations of both indoor and outdoor origin. We used the same nationwide distributions of time-activity patterns and all non-residential indoor microenvironmental PM_2.5_ concentrations from Scenario 1 because we are not aware of any robust regional data sets for these parameters. However, given that the Scenario 1 analysis demonstrated the sensitivity of the model to assumptions for residential exposures, and given that other air pollution risk assessments have shown the utility of using geographically varying population demographics and mortality rates [38, 42], we consider Scenario 2 a reasonable, albeit somewhat limited, attempt to construct a national mortality estimate using more granular input data.

Scenario 2 uses regional estimates of residential indoor PM_2.5_ concentrations of indoor origin and ambient infiltration factors recently made using a nationally representative set of combined residential energy and indoor air quality (REIAQ) models for non-smoking US residences [89]. Briefly, the REIAQ model set combined building energy models with dynamic pollutant mass balance models to estimate the hourly concentrations of a number of pollutants of indoor and outdoor origin, including PM_2.5_, in a total of 3971 individual home models in 19 cities that are estimated to represent ~80% of the US housing stock as of approximately the early 2000s. The model set assumed cooking was the primary indoor PM_2.5_ source and assumed the same generation rates and cooking frequency for all homes. The model set also accounted for historical outdoor PM_2.5_ concentrations and modeled infiltration air exchange rates, window opening behaviors, and forced air heating and cooling system runtimes based on historical outdoor environmental conditions combined with a building physics model. We used these modeled results for the regionally varying annual average residential indoor PM_2.5_ concentrations of indoor origin (i.e., Δ*C*_*PM2.5,IG,residences*_) in conjunction with regional distributions of ambient PM_2.5_ infiltration factors combined with regional distributions of outdoor PM_2.5_ concentrations for the year 2012 from EPA [82] to generate estimates of Δ*C*_*PM2.5,AG,residences*_ in each of the 19 modeled cities. We used the infiltration factor approach (rather than using values of Δ*C*_*PM2.5,AG,residences*_ directly from REIAQ) because the model set is weighted more heavily toward homes in cities with higher ambient PM_2.5_ concentrations than rural areas, while the EPA outdoor concentration data are more broadly applicable to the rest of the population.

We grouped the REIAQ model outputs for each of the 3971 home models into nine US census divisions and calculated a population-weighted annual average and SD for Δ*C*_*PM2.5,IG,residences*_ and infiltration factors (*F*_*inf*_) across all homes in each division (Table 1). We fit beta and lognormal distributions to summary statistics of infiltration factors and indoor PM_2.5_ concentration of indoor origin, respectively, for Monte Carlo sampling from each division. For PM_2.5_ of ambient origin, we used annual average (and 10th and 90th percentiles) outdoor PM_2.5_ concentration data for nine US regions reported by EPA [82]. Because the nine EPA regions group states differently than the nine US census divisions, we regrouped the EPA data by assuming that every state in an EPA region had the same annual outdoor PM_2.5_ concentration summary statistics as other states in that region. We estimated the annual average (and 10th and 90th percentile) outdoor PM_2.5_ concentration in each census division by weighting each assumed state-level summary statistic by the population in each census division. We fit lognormal distributions to the resulting estimates of annual outdoor PM_2.5_ summary statistics (means and 10th and 90th percentiles) in each division for subsequent Monte Carlo sampling.

**Table 1:** Summary of estimates for key input parameters made for each US census division for the regional analysis in Scenario 2

Census division	Mean (SD) residential indoor PM_2.5_ concentration resulting from indoor sources (µg/m^3^) [90]	Mean (SD) residential PM_2.5_ infiltration factor [90]	Mean (10th–90th percentile) outdoor PM_2.5_ concentration (µg/m^3^) in 2012 [83]	Baseline adult mortality rate in 2012, *y*_0_ (per 100,000) [88]	Over-35 adult population in 2012 (*Pop*) [88]
New England	5.78 (2.14)	0.50 (0.08)	9.22 (7.25–11.2)	1429.5	8,209,960
Middle Atlantic	5.84 (2.01)	0.50 (0.08)	9.22 (7.25–11.2)	1475.6	22,655,120
East North Central	5.66 (1.91)	0.46 (0.09)	9.98 (8.74–11.43)	1576.8	25,115,038
West North Central	5.69 (1.86)	0.45 (0.09)	9.40 (7.74–11.16)	1566.0	10,965,126
South Atlantic	6.47 (2.07)	0.40 (0.08)	8.86 (6.90–10.66)	1481.7	33,363,675
East South Central	6.81 (1.94)	0.40 (0.08)	9.82 (8.34–11.37)	1759.6	9,999,343
West South Central	4.80 (1.54)	0.46 (0.09)	9.61 (8.01–11.14)	1449.3	18,513,908
Mountain	5.38 (1.82)	0.49 (0.09)	7.95 (6.03–10.25)	1332.1	11,481,569
Pacific	6.30 (2.03)	0.46 (0.08)	9.06 (6.01–12.88)	1241.2	26,212,977

We then ran the 10,000 iteration Monte Carlo analysis 9 times—one for each census division—with over-35 adult mortality rates and populations [87] (also shown in Table 1) to yield estimates of total mortality and distributions of the different microenvironmental exposure contributions in each division. We summed the median total mortality estimates from each census division to generate an estimate of the national mortality burden associated with total PM_2.5_ exposure. We estimated the mortality burden attributable to each microenvironment and source type using the average fractional exposure contributions multiplied by the best estimate (i.e., median) total mortality, similar to Scenario 1.

## Results and Discussion

### Scenario 1: Nationwide estimates based primarily on prior field studies

**Fig. 1:**
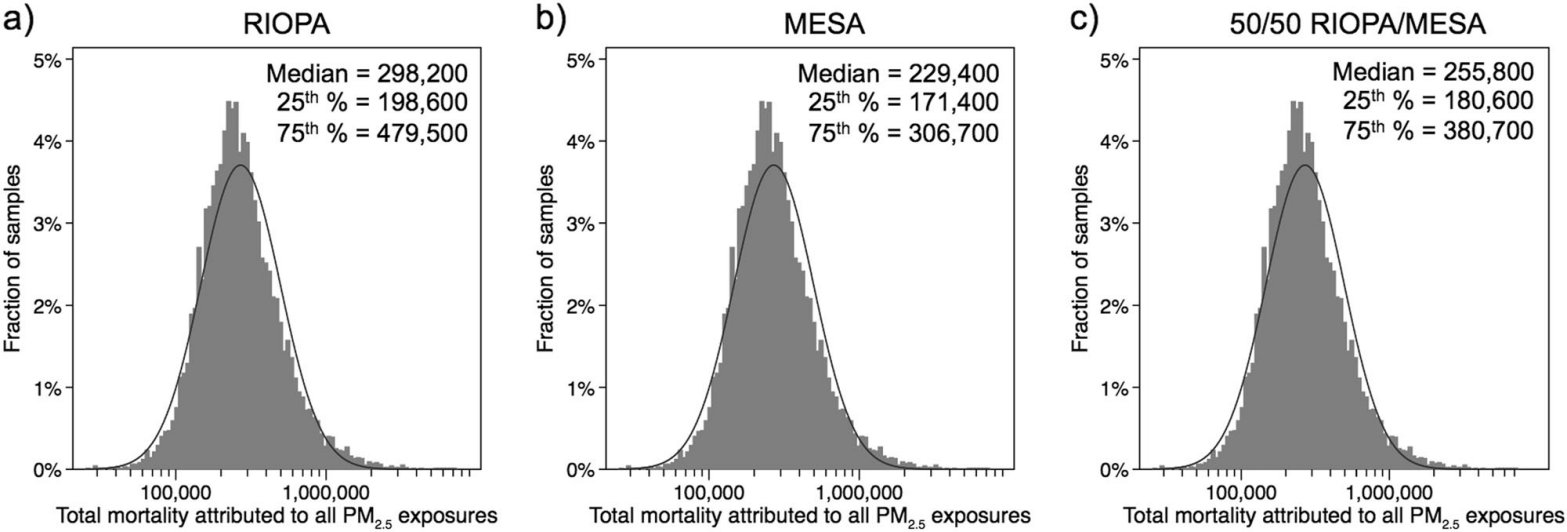
Frequency distributions of the total annual US PM_2.5_ mortality burden estimated by Monte Carlo simulations of microenvironmental exposures to PM_2.5_ of both indoor and outdoor origin using three cases in Scenario 1, including sampling residential indoor concentrations from: **a** RIOPA-only, **b** MESA-only, and **c** from RIOPA and MESA equally (i.e., 50/50 RIOPA/MESA). The approximate curve fit is a lognormal distribution and summary statistics (median and interquartile range) are provided in units of deaths per year

The resulting distributions of estimates of the annual US mortality burden of total PM_2.5_ exposure in 2012 attributable to both indoor and outdoor sources in all microenvironments combined using assumptions in Scenario 1 are shown in Fig. 1. Results for all three RIOPA and MESA sampling approaches were approximately lognormally distributed with a Shapiro–Wilk test statistic (*W)* > 0.98 and *p* < 0.00001 on the log-transformed values for each case. We consider the median values as our most likely estimate of the total mortality burden of all PM_2.5_ exposures for each scenario, with an interquartile range (IQR, or 25th to 75th percentiles) serving as a measure of the most reasonable bounds of the central estimate. The median (IQR) estimate of the total mortality associated with all PM_2.5_ exposures for each scenario were ~298,200 (198,600–479,500), ~229,400 (171,400–306,700), and ~255,800 (180,600–380,700) deaths for the 100% RIOPA, 100% MESA, and 50%/50% RIOPA/MESA scenarios, respectively. These estimates would mean that aggregate PM_2.5_ exposures accounted for between 9 and 12% of the total number of adult deaths over the age of 35 in 2012.

**Fig. 2:**
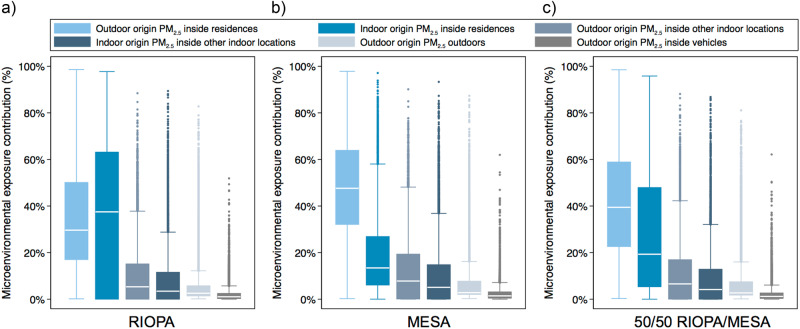
Distributions of the estimated contributions of microenvironmental exposures to PM_2.5_ of indoor and outdoor origin to total PM_2.5_ exposures across the US population using the three Scenario 1 cases: sampling residential indoor concentrations from **a** RIOPA-only, **b** MESA only, and **c** RIOPA and MESA equally (i.e., 50/50 RIOPA/MESA). Boxes represent 25th and 75th percentile values (i.e., interquartile range, or IQR); horizontal line represents median values; whiskers represent upper and lower adjacent values (i.e., 50% beyond the IQR)

Distributions of the estimated fractional exposure contributions from indoor and outdoor sources in each microenvironment modeled in Scenario 1 are shown in Fig. 2. In each of the three RIOPA/MESA cases, residential PM_2.5_ exposure to indoor and outdoor sources combined was the dominant exposure, accounting for 70% of the total PM_2.5_ exposure across all three scenarios, on average. Residential exposure accounted for an average of ~67% of the total exposure to PM_2.5_ of outdoor origin across the three scenarios, followed by an average of ~17% of outdoor origin exposure attributed to other indoor environments, with direct outdoor exposure accounting for only ~12% of all outdoor-origin exposure, on average.

In both the MESA-only and the combined RIOPA/MESA 50/50 scenarios, residential exposure to PM_2.5_ of outdoor origin dominated total exposure, accounting for an average of 48 and 42% of total exposure in the MESA-only and 50/50 combined scenarios, respectively. Residential exposure to PM_2.5_ of indoor origin was the second largest contributor to total exposure in these two scenarios, ranging from an average of 19 to 28% of total exposure in the MESA-only and 50/50 combined scenarios, respectively. Conversely, the largest contributor to total exposure in the RIOPA-only scenario was residential exposure to PM_2.5_ of indoor origin (average of 37%) followed by residential exposure to PM_2.5_ of outdoor origin (average of 36%). Given the wide ranges of exposure contributions generated by sampling from RIOPA and MESA separately, and given the large differences in the two study designs and findings, we expect the combined 50/50 RIOPA/MESA sampling approach to yield the most plausible nationwide exposure estimates of the three approaches in Scenario 1. Thus, we use only the combined 50/50 RIOPA/MESA study results from Figs. 1 and 2 to estimate the likely mortality burden associated with microenvironmental exposure to PM_2.5_ of indoor and outdoor origin in Scenario 1 (Table 2).

**Table 2:** Mean, standard deviation (SD), and interquartile range (IQR: 25th to 75th percentiles) of the estimated contributions of indoor and outdoor sources in each microenvironment to total PM_2.5_ exposures and the estimated associated US mortality burden in Scenario 1 (50/50 RIOPA/MESA)

Outdoor or indoor sources	Microenvironment	Mean fraction of total PM_2.5_ exposure ± SD [IQR]	Mean estimate of annual deaths attributed to total PM_2.5_ exposure ± SD [IQR]
Due to PM_2.5_ of outdoor origin	Residences	42.1 ± 23.8%	107,700 ± 61,000
		[22.6–58.9%]	[57,800–150,600]
	Other indoor locations	10.9 ± 12.9%	28,000 ± 33,000
		[0–16.9%]	[100–43,300]
	Vehicles	2.4 ± 3.9%	6100 ± 9900
		[0.3–2.6%]	[900–6700]
	Outdoor	7.3 ± 11.1%	18,800 ± 28,600
		[1.8–7.5%]	[4,500–19,100]
Total outdoor contribution		62.7 ± 25.2%	160,500 ± 40,400 [63,300–219,600]
Due to PM_2.5_ of indoor origin	Residences	28.1 ± 26.3%	72,000 ± 67,300
		[5.3–47.9%]	[13,700–122,600]
	Other indoor locations	9.1 ± 12.6%	23,300 ± 32,200
		[0–12.8%]	[0–32,800]
	Vehicles	n/a	n/a
		n/a	n/a
	Outdoor	n/a	n/a
		n/a	n/a
Total indoor contribution		37.3 ± 25.2%	95,300 ± 24,000 [13,700–155,400]
Total contribution		100%	255,800 [77,000–375,000]

Mortality burden estimates are based on the ‘best estimate’ of the median total mortality burden resulting from Monte Carlo sampling of the RIOPA and MESA studies combined (i.e., each sampled equally: 50/50 RIOPA/MESA)

We estimate the mortality burden associated with PM_2.5_ exposure in each microenvironment by multiplying the mean fractional exposure contribution (from Fig. 2) by the median total mortality burden of ~255,800 deaths per year for the combined 50/50 RIOPA/MESA scenario (from Fig. 1). Using this approach, we estimate that exposure to PM_2.5_ of outdoor origin across all microenvironments accounted for ~160,500 deaths in 2012 (IQR of ~63,300 to ~219,600 deaths), while exposure to PM_2.5_ of indoor origin across all microenvironments accounted for ~95,300 deaths (IQR of ~13,700 to ~155,400). Our estimate of the mortality burden attributable to outdoor sources is between the ~120,000 and ~200,000 deaths in 2010 estimated by Fann et al. (2017) [42] using RR estimates and response functions from Krewski et al. (2009) [5] and Nasari et al. (2016) [45], respectively. However, our estimate is almost twice the ~88,400 deaths in 2015 estimated by Cohen et al. (2017) [55] largely because of the threshold concentration used (i.e., zero compared to a uniform distribution between 2.4 and 5.8 µg/m^3^) and also because of the use of a different model form and associated effect estimates that are not modified to account for microenvironmental exposure to outdoor-origin PM_2.5_. Both issues are explored in more detail in Scenario 3 in the SI.

In the combined 50/50 RIOPA/MESA scenario, we estimate that the largest contributor to PM_2.5_-associated mortality is residential indoor exposure to PM_2.5_ of outdoor origin, accounting for an estimated ~107,700 deaths annually (IQR of ~57,800 to ~150,600). The next largest contributor is residential indoor exposure to PM_2.5_ of indoor origin, accounting for an estimated ~72,000 deaths annually (IQR of ~13,700 to ~122,600). Indoor exposure to PM_2.5_ of indoor and outdoor origin in other indoor locations is estimated to account for ~23,300 (IQR of ~0 to ~32,800) and ~28,000 (IQR of ~100 to ~43,300) deaths annually, respectively. Finally, outdoor exposure to PM_2.5_ of outdoor origin is estimated to account for only ~18,800 (IQR of ~4,500 to ~19,100) deaths annually. Overall, these results demonstrate the importance of indoor environments, and particularly residential indoor environments, in governing human exposure to PM_2.5_ of both indoor and outdoor origin, and provide novel estimates of the potential magnitude of the nationwide mortality burden associated with these exposures.

### Scenario 2: Nationwide estimate based on regional model outputs

Table 3 shows estimates of regional and total mortality associated with microenvironmental PM_2.5_ exposures resulting from the regional model application (Scenario 2). The median (IQR) estimate of the total mortality associated with all PM_2.5_ exposures across all microenvironments and sources was ~281,800 (159,700–359,300), which places Scenario 2 approximately between the RIOPA-only and 50/50 RIOPA/MESA cases from Scenario 1. Exposure to PM_2.5_ of outdoor and indoor origin in all microenvironments was estimated to account for ~139,500 deaths (IQR of ~69,600 to ~177,900) and ~142,300 deaths (IQR of ~90,100 to ~181,400) in 2012, respectively. The relative contributions of indoor and outdoor PM_2.5_ sources to total mortality are approximately equal, largely because of the use of relatively high indoor concentrations (similar to the RIOPA-only approach in Scenario 1) and relatively low residential infiltration factors that were estimated in the REIAQ model set. Accordingly, residential indoor PM_2.5_ of indoor origin is estimated to be the single dominant contributor to the total mortality burden in Scenario 2, followed by residential indoor PM_2.5_ of outdoor origin.

**Table 3:** Estimates of regional and total mortality associated with microenvironmental exposures to PM_2.5_ of indoor and outdoor origin in 2012 resulting from the regional Monte Carlo procedure (Scenario 2)

Census division	Percentile	Total mortality	Mortality associated with PM_2.5_ of ambient origin					Mortality associated with PM_2.5_ of indoor origin		
			Inside residences	Other indoors	Vehicles	Outdoor	Total	Inside residences	Other indoors	Total
New England	Median	13,900	4500	1400	300	1000	7200	5500	1200	6700
	25th %	7900	3400	0	100	200	3700	4200	0	4200
	75th %	17,600	5600	2200	300	1000	9100	6900	1600	8600
Middle Atlantic	Median	39,600	12,800	4100	900	2600	20,400	15,900	3300	19,300
	25th %	22,700	9800	0	100	700	10,600	12,200	0	12,200
	75th %	50,300	15,800	6300	1000	2600	25,700	19,900	4600	24,500
East North Central	Median	47,100	15,100	5200	1100	3300	24,800	18,300	4000	22,300
	25th %	26,200	11,300	0	200	900	12,500	13,800	0	13,800
	75th %	60,200	18,800	8200	1200	3400	31,700	23,000	5500	28,500
West North Central	Median	19,700	6100	2100	400	1300	9900	8100	1700	9800
	25th %	11,100	4600	0	100	400	5000	6100	0	6100
	75th %	25,100	7500	3300	500	1300	12,600	10,100	2400	12,500
South Atlantic	Median	56,200	14,600	5700	1200	3700	25,300	26,100	4800	30,900
	25th %	32,400	10,800	0	200	1000	12,000	20,400	0	20,400
	75th %	71,600	18,200	9000	1400	3800	32,500	32,400	6700	39,200
East South Central	Median	21,600	5800	2300	500	1500	10,000	9800	1800	11,600
	25th %	12,500	4400	0	100	400	4900	7700	0	7700
	75th %	27,500	7100	3700	600	1500	12,800	12,100	2500	14,600
West South Central	Median	29,300	9800	3300	700	2100	16,000	10,600	2700	13,300
	25th %	15,800	7300	0	100	600	8000	7800	0	7800
	75th %	37,700	12,400	5300	800	2200	20,600	13,400	3700	17,100
Mountain	Median	16,000	4800	1600	300	1000	7800	6700	1500	8200
	25th %	9000	3700	0	100	300	4000	5100	0	5100
	75th %	20,400	6000	2500	400	1000	9800	8400	2100	10,500
Pacific	Median	38,500	11,100	3800	800	2500	18,200	17,000	3300	20,300
	25th %	22,000	8300	0	100	600	9000	13,000	0	13,000
	75th %	48,900	13,800	5900	900	2400	23,000	21,300	4600	25,900
Total	Median	281,800	84,700	29,500	6200	19,100	139,500	118,000	24,400	142,300
	25th %	159,700	63,500	0	1100	5000	69,600	90,100	0	90,100
	75th %	359,300	105,200	46,400	7100	19,200	177,900	147,600	33,800	181,400

Total mortality in Scenario 2 is driven largely by PM_2.5_ exposures in the most populated census divisions: South Atlantic, East North Central, Middle Atlantic, and Pacific. The East South Central census division had the highest estimated mortality associated with PM_2.5_ per capita because of relatively high residential indoor concentrations resulting form indoor sources combined with the highest baseline adult mortality rate in 2012. The lowest per capita mortality estimate was in the Mountain census division, with moderate residential indoor PM_2.5_ concentrations and a moderate baseline mortality rate. Regional differences in Δ*C*_*PM2.5,IG,residences*_ were driven variations in air exchange rates [89] and system runtimes (which primarily affects particle filtration [90]).

**Fig. 3:**
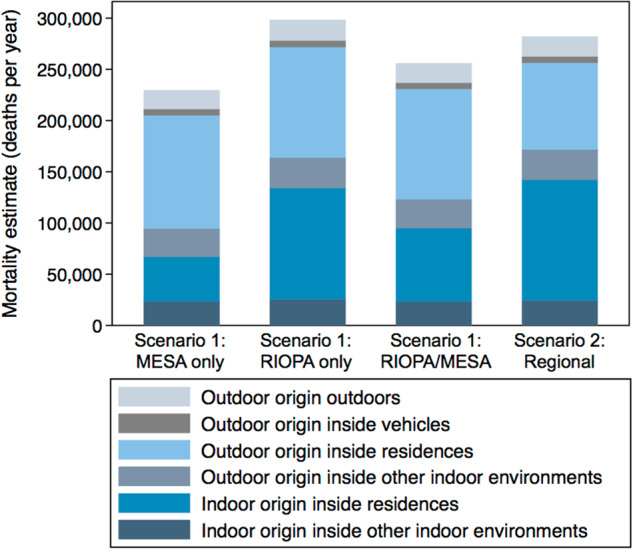
Best estimates of the number of annual deaths in the US associated with exposure to PM_2.5_ of indoor and outdoor origin in each microenvironment in Scenarios 1 and 2

Best estimates of the total mortality burden associated with PM_2.5_ exposure in the US made using the assumptions in Scenarios 1 and 2, as well as the contribution of each microenvironmental and source-specific exposure, are shown in Fig. 3 for direct comparison. Although the magnitude of total mortality varies in each scenario, best estimates consistently range from ~230,000 to ~300,000 deaths in 2012. Residential exposures to PM_2.5_ from indoor sources drive the vast majority of variability in each case, suggesting that a better understanding of the nationwide contribution of indoor sources to total exposure are needed, as is a better understanding of the toxicity of indoor sources.

### Limitations

One obvious assumption in this work is that the observed relationships between outdoor PM_2.5_ concentrations and mortality in the epidemiology literature are indeed causal and that the underlying exposure-response functions and effect estimates accurately reflect a causal and quantifiable relationship [91–94]. Further, the framework assumes that the exposure-response function in Eq. 1 (i) has no threshold PM_2.5_ concentration below which additional mortality does not occur [83–86] and (ii) appropriately describes the shape of the observed mortality responses from prior epidemiology studies [95]. Additionally, we do not make any modifications to the exposure-response function and effect estimates based on the magnitude of PM_2.5_ exposure concentrations or varying chemical constituents of PM_2.5_, although there is some evidence that both of these adjustments may be warranted [96–100]. Moreover, the framework assumes that there is no double counting of the health effects of indoor PM_2.5_ sources. We consider this a reasonable assumption because most studies have reported relatively low correlations between personal and ambient PM_2.5_ concentrations (i.e., *R*^2^ < 0.3) [13, 69], but the potential for ambient PM_2.5_ mortality effect estimates resulting from epidemiology cohort studies including an inherent but un-quantified indoor contribution remains.

Another obvious assumption and potential limitation in this work is that we assume that the modified exposure-response endpoint effect estimates for mortality associated with PM_2.5_ from both indoor and outdoor sources are the same, and that there are no changes in PM_2.5_ toxicity that occur due to size-resolved aerosol dynamics that govern the particle infiltration and persistence process. Although some studies have suggested that particles of outdoor origin may be more harmful than indoor-generated particles [59, 60], other studies have shown that indoor-generated fine particulate matter is at least as toxic as outdoor particulate matter [61], if not more [62]. However, there is a tremendous lack of data to support or reject either assumption at this time. Given the lack of data on mortality endpoints from various indoor and outdoor PM_2.5_ sources, we consider this a reasonable assumption for this exploratory analysis. This same assumption also has precedent in a number of other recent studies in the literature that have evaluated mortality endpoints associated with indoor and outdoor PM_2.5_ sources [46–48]. Additionally, there is mounting evidence from air filter intervention studies in homes that reducing indoor PM_2.5_ concentrations (comprising a mixture of both indoor and outdoor sources) can lead to improvements in some biomarkers and other clinical measures that are associated with both short-term and long-term cardiovascular health endpoints [101–107].

There are also several assumptions implicit in our approach to modifying health endpoint effect estimates (*β*) to account for the underlying exposures to PM_2.5_ of outdoor origin that likely occurred in the original epidemiology populations from which effect estimates are derived. First, we assumed that the distributions of activity patterns and residential building characteristics (i.e., infiltration factors) that we used match both the general population and the epidemiology cohort populations, although this may not be true. For example, elderly populations who are more susceptible to adverse effects associated with PM_2.5_ exposure tend to spend more time indoors than the general population. Second, we did not consider some potential non-linear effects of various parameters including potential covariance of infiltration factors and ambient PM_2.5_ as well as occupancy and indoor particle generation. Third, we assumed that the human activity patterns reported in NHAPS [17] are still valid in 2012, even though data were collected in 1992–1994.

Despite the large uncertainties associated with this work, the exposure attribution and mortality burden estimates clearly demonstrate the importance of considering indoor microenvironments in PM_2.5_ exposure assessments and epidemiology studies. They also illustrate the potential magnitude and reasonable bounds of the mortality burden potentially associated with microenvironmental exposures to PM_2.5_ of both indoor and outdoor origin. Results also demonstrate that efforts to reduce the US PM_2.5_ associated mortality burden should at least consider indoor pollutant control in addition to controlling outdoor sources. This model framework can also be used for high-level policy analysis of the costs and benefits of reducing exposures to PM_2.5_ of indoor and outdoor origin through various interventions (e.g., source control, air purifiers, changing infiltration/ventilation across the building stock, etc.).

This work intentionally focuses solely on non-smoking homes; further model applications could include incorporating data on smoking rates and contributions to indoor PM_2.5_ concentrations. This work also highlights the need for several areas of research to improve these estimates and reduce uncertainty. For example, a better understanding of how outdoor PM_2.5_ infiltration factors vary geographically and by different building types is needed to more accurately characterize outdoor PM_2.5_ exposures for epidemiology studies. Additionally, a better understanding of the toxicity of both indoor and outdoor origin PM_2.5_ is needed, including characterizing the toxicity of a wide variety of typical indoor sources and also characterizing how the size-resolved dynamics of the outdoor PM_2.5_ infiltration process may affect the toxicity of PM_2.5_ of outdoor origin in indoor environments.

## Electronic supplementary material

Supplemental Information
