# Supplementary material for: A framework for estimating the US mortality burden of fine particulate matter exposure attributable to indoor and outdoor microenvironments
Source: J Expo Sci Environ Epidemiol. 2018 Dec 5;30(2):271–84. doi: 10.1038/s41370-018-0103-4 (PMC7039807; doi:10.1038/s41370-018-0103-4)
Supplement: Supplementary file 1 — Supplemental Information [file 41370_2018_103_MOESM1_ESM.docx]

**Supplementary Information for:**

**A Framework for Estimating the U.S. Mortality Burden of Fine Particulate Matter Exposure Attributable to Indoor and Outdoor Microenvironments**

Parham Azimi, Ph.D.,^1^ Brent Stephens, Ph.D.^1^

^1^Department of Civil, Architectural, and Environmental Engineering, Illinois Institute of Technology, Chicago, IL USA

**Table of contents**

Selecting appropriate exposure-response effect estimates for PM_2.5_ of outdoor origin 2

Modifying exposure-response function effect estimates for PM_2.5_ of outdoor origin 3

Scenario 1: Nationwide estimate based primarily on prior field studies 4

Scenario 3: Global Burden of Disease Integrated Exposure-Response model 9

References 14

Figures 19

Tables 20

# Selecting appropriate exposure-response effect estimates for PM_2.5_ of outdoor origin

Epidemiology studies that have investigated associations between premature all-cause mortality and long-term outdoor PM_2.5_ concentrations in the U.S. have reported various relative risks (RR) commonly ranging from 6% per 10 µg/m^3^ (95% CI: 2% to 11%) to 26% per 10 µg/m^3^ (95% CI: 8% to 47%).^1–9^ The differences in magnitudes of RR from each study are attributable to a combination of varying cohort population demographics, population susceptibility, outdoor PM_2.5_ sources and compositions, and other factors including those that affect infiltration and persistence in residential indoor environments where people spend most of their time.^10–16^ Despite this variability, it is common to assume that all outdoor PM_2.5_ is equally potent in producing premature mortality regardless of geographic location or other factors for these kinds of population-level analyses.^9^ Therefore, we utilize a central pooled estimate of RR for the increase in long-term all-cause mortality associated with outdoor PM_2.5_ concentrations in the U.S. of 7.3% per 10 µg/m^3^ (95% CI: 3.7% to 11%) as reported in a recent quantitative meta-analysis of outdoor PM_2.5_ concentration-response effect estimates.^17^ This estimate is similar in magnitude to, albeit somewhat higher than, another recent pooled effect estimate of 6% (95% CI: 4% to 8%) for all-cause mortality made using a meta-analysis of studies from both the U.S. and Europe.^18^ We convert the pooled RR estimate of 1.073 per 10 µg/m^3^ to an effect estimate (i.e., *β_PM2.5_*) of 0.0070 (95% CI: 0.0036 to 0.0104), where *β_PM2.5_ =* ln(RR)/10.^19^ We fit a Weibull distribution to these reported values in MATLAB, resulting in a mean (±SD) value of *β_PM2.5_* = 0.0070 (±0.0016) per µg/m^3^ with distribution shape factors of α = 0.765 and β = 4.95. A Weibull distribution was used because it yields a distribution that is very close to normal in shape, but does not produce any negative values.

# Modifying exposure-response function effect estimates for PM_2.5_ of outdoor origin

Average times spent in each microenvironment were taken directly from the 1992-1994 National Human Activity Pattern Survey (NHAPS),^20^ as described in the main text. Average outdoor PM_2.5_ infiltration factors for each microenvironment (i.e., *F_j_*) were culled from the existing literature as follows. We assumed the average ambient PM_2.5_ infiltration factor inside U.S. residences was 0.59 according to the mean value reported in a recent review of 17 studies spanning over 1000 U.S. homes.^21^ We assumed the average PM_2.5_ infiltration factor in all other indoor locations other than residences was 0.49 based on the average modeled indoor concentrations of outdoor origin from a simulation study of small and medium commercial buildings that were designed to reasonably represent the U.S. office building stock.^22^ Briefly, we used MATLAB to fit Weibull and lognormal distributions to their reported percentiles of indoor and outdoor PM_2.5_ concentrations, then used Monte Carlo simulations with 20,000 iterations to build a distribution of infiltration factors based on these two distributions (i.e., *F_inf_* = *C_in_*/*C_out_* in the absence of indoor sources). Here we use the mean value of the resulting distribution. While this analysis ignored other non-residential buildings, we are not aware of other robust data sets on infiltration factors in non-office non-residential buildings in the U.S. However, an infiltration factor of 0.49 is reasonably in range with those reported in other non-residential buildings in Europe, including workplaces in Finland^23^ and schools in Germany^24^ and Spain.^25^ For vehicles, we assumed an average ambient PM_2.5_ infiltration factor of 0.43, assuming personal vehicles operate 50% of the time in recirculated air mode and 50% of the time in outdoor air ventilation mode with infiltration factors of 0.25 and 0.61, respectively.^26^ Finally, we assumed that people are exposed to 100% of PM_2.5_ of outdoor origin when they spend time outdoors (i.e., *F_outdoor_* = 1).

# Scenario 1: Nationwide estimate based primarily on prior field studies

Here we describe all relevant model inputs and data sources that were used for the nationwide PM_2.5_ mortality estimate made in Scenario 1. To characterize what we assume to be nationally representative time-activity patterns, Table S1 shows stair-step distribution characteristics for the amount of time spent in each of the four microenvironments considered herein, which we transcribed from Figure 3 in Klepeis et al. (2001).^20^ Values for the fraction of time spent in residences, vehicles, and outdoors were all sampled directly from the stair-step distributions in Table S1, while the time spent in other indoor locations was estimated by subtracting the sum of times spent in these three microenvironments from a total of 1440 minutes (i.e., 24 hours).

To characterize indoor PM_2.5_ concentrations of indoor origin in all non-residential indoor microenvironments, we assumed an arithmetic mean (± SD) of 4.18 ± 4.98 µg/m^3^, which was suggested for the range of indoor PM_2.5_ concentrations resulting from general office work environments in Finland.^23^ We constructed a lognormal distribution that resulted in the same arithmetic mean ± SD to avoid negative values while matching the same summary statistics (GM = 2.69 µg/m^3^ and GSD = 2.56). Although this approach is limited to office buildings in Finland, we are not aware of other studies that have similarly quantified the indoor and ambient contributions to indoor concentrations in non-residential buildings in the U.S. We also consider it reasonable to assume the same values for all non-residential indoor microenvironments regardless of building function based on the close similarities in indoor PM_2.5_ concentrations reported in a recent study of a wide variety of small and medium sized commercial environments in California.^27^

To characterize indoor PM_2.5_ concentrations of outdoor origin in non-residential indoor environments, we sampled from the same beta distribution of infiltration factors in small and medium U.S. commercial buildings that we constructed previously from Ben-David et al. (2017).^22^ Shape factors for the infiltration factor beta distribution in MATLAB were: α = 1.82 and β = 1.91 (Figure S1). Similarly, to characterize PM_2.5_ concentrations of outdoor origin inside vehicles, we sampled equally from two beta distributions fit to reported summary statistics of infiltration factors measured in vehicles operating half the time in recirculated air mode and half the time in outdoor air ventilation mode, with mean ± SD infiltration factors of 0.25±0.12 and 0.61±0.17, respectively.^26^ Shape factors for the assumed beta distributions were α = 2.98 and β = 8.72 for recirculating systems and α = 4.32 and β = 2.73 for outdoor air ventilation (Figure S1). We also introduced an in-vehicle exposure modification factor to Equation 3-b to account for near-road PM_2.5_ concentrations that are 22% higher, on average, than central site monitor or background levels.^28^ This factor is not incorporated directly into the model framework but is applied uniformly to each model iteration during application. We assumed there are no indoor sources of PM_2.5_ inside vehicles because the focus is on non-smoking microenvironments and it is reasonable to assume there are minimal other PM_2.5_ sources inside most vehicles.

To characterize indoor PM_2.5_ concentrations from both indoor and outdoor sources in residences, we rely on two of the largest data sources for field measurements of which we are aware: the Relationship of Indoor, Outdoor and Personal Air (RIOPA) study^29^ and the Multi-Ethnic Study of the Atherosclerosis and Air Pollution (MESA Air).^30,31^ The RIOPA study sampled indoor and outdoor PM_2.5_ concentrations concurrently for 48 hours in 212 non-smoking residences in three U.S. cities.^32^ Similarly, MESA Air measured indoor and outdoor PM_2.5_ concentrations concurrently over a 2-week period in 208 homes in warm seasons and 264 homes in cold seasons in seven U.S. cities. Both studies are unique in that they included large sample sizes of homes in multiple U.S. cities and also reported distributions of PM_2.5_ infiltration factors, which can be either directly or indirectly used to estimate the relative contributions of both indoor and outdoor sources to indoor PM_2.5_ concentrations in the sample residences. Because the two studies differed in their population demographics and geographic locations, we use them to conduct three versions of Scenario 1, including sampling input parameters affecting indoor concentrations of PM_2.5_ of indoor and outdoor origin from: (a) RIOPA only, (b) MESA only, and (c) equally from both RIOPA and MESA. For Scenarios 1a and 1b, we assume that either RIOPA or MESA is generally representative of the entire U.S. residential building stock. Because this may not be a valid assumption for either study, Scenario 1c assumes that, when sampled together with equal weighting, the two studies are more generally representative than either study alone.

For the RIOPA-only scenario, we estimated distributions of annual average residential indoor PM_2.5_ concentrations of outdoor origin for the year 2012 by sampling from distributions of infiltration factors reported in the study homes and multiplying them by samples drawn from distributions of the U.S. annual average outdoor PM_2.5_ concentration for 2012, as described in the main text. Meng et al. (2005) modeled the distributions of outdoor PM_2.5_ infiltration factors of a subset of 114 of the study homes that had one complete set of 48-hour measurements, resulting in a mean (±SD) of 0.54 ± 0.16.^33^ We fit a beta distribution to the reported mean ± SD infiltration factors from RIOPA, resulting in shape factors of α = 4.70 and β = 4.00 (Figure S1).

To obtain distributions of the indoor PM_2.5_ concentration resulting from indoor sources alone, lognormal distributions were first fit to match the reported means and standard deviations of indoor and outdoor PM_2.5_ concentrations from the RIOPA study, as shown in Table S2. Next, distributions for the indoor PM_2.5_ concentration resulting from only indoor sources in the RIOPA residences were estimated using Equation S1 combined with sampling from lognormal distributions for indoor and outdoor PM_2.5_ concentrations and the beta distribution for outdoor PM_2.5_ infiltration factors. Using this approach, we make the necessary assumption that Δ*C_PM2.5,AG,residences_* varies over time (i.e., from year to year) because ambient concentrations vary over time, and that infiltration factors are constant over time. Similarly, we assume that indoor emission sources, and thus Δ*C_PM2.5,IG,residences_*, are also constant over time.

| $C_{PM2.5,IG, residence,in}=C_{PM2.5, residence}-(C_{PM2.5, out}*F_{residence})$ | (S1) |
| --- | --- |

where $C_{PM2.5,IG, residence}$ is the indoor PM_2.5_ concentration of indoor origin estimated in the RIOPA residences (µg/m^3^); $C_{PM2.5, residence}$ is a sampled value of the indoor PM_2.5_ concentration in RIOPA residences based on the distributions fit to data reported in Meng et al. (2005)^32^ (µg/m^3^); $C_{PM2.5, out}$ is a sampled value of the simultaneous outdoor PM_2.5_ concentration in the RIOPA study based on the distributions fit to data reported in Meng et al. (2005)^32^ (µg/m^3^); and $F_{residence}$ is a sampled value of the corresponding outdoor PM_2.5_ infiltration factor for the same RIOPA residences based on the distributions fit to data reported in Meng et al. (2005)^33^ (-). Using this approach, we estimate that the average contribution of indoor sources to indoor PM_2.5_ concentrations in the RIOPA study was ~9.5 µg/m^3^, or ~63% of the total indoor PM_2.5_ concentration, with ~37% coming from outdoor sources, on average.

For the MESA Air scenario, we also created distributions of annual average residential PM_2.5_ concentrations of indoor and outdoor origin for 2012 by sampling from distributions of infiltration factors and indoor PM_2.5_ concentrations that were attributable to indoor sources, as both were reported directly by Allen et al. (2012).^34^ Similar to RIOPA, a beta distribution was fit to the reported mean ± SD infiltration factors (0.62±0.21), with shape factors of α = 2.69 and β = 1.65 (Figure S1). We also fit lognormal distributions to the summary statistics for indoor PM_2.5_ concentrations that were attributable to indoor sources (i.e., $C_{PM2.5, residence,in}$) in Allen et al. (2012), using the average of cold and warm seasons to represent an assumed annual average (Table S2). Using this approach, the average contribution of indoor sources to indoor PM_2.5_ concentrations in MESA Air was ~2.76 µg/m^3^, or only ~30% of the total indoor PM_2.5_ concentration, on average, with ~70% coming from outdoor sources. Clearly, the MESA and RIOPA scenarios represent very different assumptions for the relative contributions of indoor and ambient sources to residential indoor PM_2.5_ concentrations. For the combined 50/50 RIOPA/MESA scenario, we sampled from each of the generated distributions equally.

In an attempt to verify our model framework, we also repeated Scenario 1 with inputs modified to match those used by Fann et al. (2017)^35^ to estimate the ambient PM_2.5_ mortality burden in the US in 2010. In theory, our model framework should be able to reasonably recreate mortality estimates made using only outdoor PM_2.5_ concentrations as surrogates for exposure (i.e., the approach used in Fann et al. 2017) since outdoor concentrations should be appropriately re-assigned in the model as exposure estimates in various microenvironments. We re-ran the analysis using the following inputs for the US adult population 35 years and older in 2010: *Pop* = 162,828,035; *y*_0_ = 1450.8 per 100,000 persons per year, *β_PM2.5_* = 0.583 mean with SD of 0.096 (and a beta distribution fit through the mean and SD), population-weighted average outdoor PM_2.5_ concentration of 8.8 µg/m^3^ (taken directly from Fann et al. 2017), and a threshold outdoor PM_2.5_ concentration of zero. Using the model framework with these assumptions and keeping all other assumptions from the 50/50 RIOPA/MESA combined scenario the same, our best estimate (i.e., median value) of the total annual mortality burden associated with exposure to PM_2.5_ of both indoor and outdoor origin across all microenvironments in 2010 was ~198,100 deaths (IQR of ~149,600 to ~281,100). The relative microenvironmental exposure contributions were similar to those in Figure 2c in the main text, and our best estimate of the mortality burden associated with exposure to PM_2.5_ of outdoor origin summed across all microenvironments was ~124,500 deaths (IQR of ~49,400 to ~169,700). The result was very similar to the central estimate of ~120,000 (95% CI of 83,000 to 160,000) annual deaths made by Fann et al. (2017),^35^ which serves as a reasonable check on the validity of our modeling approach and the accuracy of our results, at least for PM_2.5_ of ambient origin, even though we consider much less detail in geographical variations in population and ambient exposures than Fann et al. (2017). For comparison, the estimated mortality burden associated with indoor PM_2.5_ sources in this scenario was ~73,600 deaths (IQR of ~10,700 to ~118,300).

# Scenario 3: Global Burden of Disease Integrated Exposure-Response model

The Global Burden of Disease (GBD) study and others^36–40^ have used the integrated exposure-response (IER) model developed by Burnett et al. (2014),^41^ which estimates the relative risk (RR*_i_*) of long-term exposure to a wide range of PM_2.5_ concentrations and sources for multiple causes of mortality using Equation S2.

| ${RR}_{i}=1+\alpha_{i}\left[ 1-e^{{-\gamma_{i}(C-C_{0})}^{\delta_{i}}} \right]$ for *C* > *C*_0_ | (S2-a) |
| --- | --- |
| ${RR}_{i}=1$ for *C* ≤ *C*_0_ | (S2-b) |

where *C* is the exposure concentration (μg/m^3^), *C*_0_ is the concentration below which there is an assumption of no additional risk (μg/m^3^), *i* is one of five causes of mortality, and *α_i_*, *γ_i_*, and *δ_i_* are statistical parameters that result from fitting the model to RR results from a large number of global epidemiological studies for each mortality endpoint. The premature mortality *M* for a given population range *j* and disease endpoint *i* is estimated for a given region using Equation S3.^36^

| $M_{i,j}={Pop}_{j}{\hat{y_{0}}}_{i,j}({RR}_{i,j}\left( C \right))-1)$ where ${\hat{y_{0}}}_{i,j}=\frac{{y_{0}}_{i,j}}{\bar{RR}_{i}}$ | (S3) |
| --- | --- |

where ${Pop}_{j}$ is the region’s population for age range *j* and ${y_{0}}_{i,j}$ is the region’s annual average disease incidence for population age range *j*, and $\bar{RR}_{i}$ is the average population-weighted relative risk for endpoint *i*, as shown in Equation S4.^36^

| $\bar{RR}_{i}=\frac{\sum_{j=1}^{N_{i}} {Pop}_{j}{RR}_{i,j}\left( C \right)}{\sum_{j=1}^{N} {Pop}_{j}}$ | (S4) |
| --- | --- |

where $N_{i}$ is the total number of age ranges for endpoint *i*.

The IER methodology was developed in part because the exposure-response function in Equation 1 in the main text is based on epidemiology cohort studies in the U.S. and Europe with outdoor PM_2.5_ concentrations typically below 30 µg/m^3^, which may not be representative for countries with much higher ambient air pollution levels^37^ or for other, higher, PM_2.5_ exposures such as secondhand- or active-smoking. Therefore, the IER methodology integrates estimates of the RR of multiple causes of mortality, including ischemic heart disease (IHD), cerebrovascular disease (stroke), chronic obstructive pulmonary disease (COPD), and lung cancer (LC) for adults over 25, as well as acute lower respiratory infection (ALRI) for children under 5, that have been associated with a wide range of PM_2.5_ exposure concentrations (i.e., from 0 to ~30,000 µg/m^3^) resulting from a variety of PM_2.5_ sources, including ambient air pollution, secondhand smoke, active smoking, and household air pollution. RR estimates are then converted to population-wide excess mortality estimates using age-specific mortality and demographic data.

As an example application of the IER approach, Cohen et al. (2015) estimated that approximately 88,400 deaths (95% CI: 66,800-115,000) were associated with outdoor PM_2.5_ exposures in the U.S. in 2015.^39^ Similarly, Apte et al. (2015) estimated that approximately 103,000 deaths (CI unknown) were associated with outdoor PM_2.5_ exposures in the U.S. in 2010.^36^ As another example, Wang et al. (2017)^42^ used the IER model – combined with look-up tables for RR values across a range of ambient PM_2.5_ concentrations from 5.8 to 410 µg/m^3^ provided by Apte et al. (2015)^37^ – to estimate the mortality burden associated with ambient PM_2.5_ exposure in high-income North America (i.e., Canada and the U.S.) to be only ~51,000 deaths in 2010. We approximate the U.S. mortality burden from this same estimate to be in the range of ~45,000 given that Canada had ~11% of the population of the U.S. in 2010. Another more recent study introduced the Global Exposure Mortality Model (GEMM), building on the IER model, for estimating global mortality associated with ambient PM_2.5_ exposures. Their estimate of excess all-cause mortality attributable to ambient PM_2.5_ in North America in 2015 was ~213,000 deaths, while the same estimate using the IER approach was only 95,000, suggesting that the IER model underestimates all-cause mortality.^43^

For comparison purposes, we re-ran a version of Scenario 1 using the IER model approach (i.e., substituting Equations S1-S4 for Equation 1 in the Monte Carlo analysis) to estimate the premature mortality for adults 35 years and older attributable to IHD, stroke, COPD, and LC. We excluded ALRI in young children because of its extremely low prevalence in the U.S. We considered one age range (i.e., 35 years and older) for COPD and LC, and ten age ranges for IHD and stroke (i.e., 35-40, 40-45, 45-50, 50-55, 55-60, 60-65, 65-70, 70-75, 75-80, and +80 years old). Table S3 summarizes the population and disease incidence for each of these age groups and disease conditions gathered from the CDC WONDER system.^44^ We used the RR look-up tables from Apte et al. (2015)^37^ similar to Wang et al. (2017)^42^ to assign a RR value for each mortality endpoint and modeled concentration. This necessitates making the assumption of a 5.8 µg/m^3^ threshold concentration, below which no additional mortality is assumed.

Using the IER approach with inputs from Scenario 1, we estimate that the total mortality associated with PM_2.5_ exposure from all sources and across all microenvironments is ~38,400 deaths annually (Table S4). We estimate that exposure to PM_2.5_ of outdoor origin across all microenvironments accounted for ~24,000 deaths in 2012 (IQR of ~9,200 to ~32,700 deaths), and exposure to PM_2.5_ of indoor origin across all microenvironments accounted for ~14,400 deaths (IQR of ~2,000 to ~22,400). Results from the IER scenario are drastically lower than results from Scenario 1 and 2 for several reasons, primarily including: (1) the use of a 5.8 µg/m^3^ threshold concentration compared to the no-threshold assumption; (2) the IER model presents a different model form and parameter fits that may yield different effect estimates from the traditional exposure-response (E-R) function and effect estimates used herein; and (3) the IER model is fit through ambient air pollution studies at the low end of the concentration range, and is thus not modified for microenvironmental exposures to PM_2.5_ of outdoor origin that would have occurred in the original cohort studies. Results for the total mortality associated with PM_2.5_ of outdoor origin from the IER scenario are also lower than previous estimates, including: just over half of that reported by Wang et al. (2017)^42^ (mostly attributed to our modification of ambient origin exposures by microenvironmental infiltration factors), and approximately one-quarter of that reported by Cohen et al. (2015)^39^ and Apte et al. (2015)^36^ (mostly attributed of the aforementioned underlying discrepancies that exist between Wang et al. and Cohen et al. and Apte et al., for reasons that are not immediately clear).

For these varied reasons, a comparison between the IER approach in Scenario 3 and the E-R approaches in Scenario 1 and 2 is not directly valid without some additional modification. Therefore, we re-ran Scenario 1 again using the original E-R model form (i.e., Equation 1 in the main text) with the following changes to provide a more appropriate comparison to the IER model: (1) we introduced a threshold concentration of 5.8 µg/m^3^ to each model iteration, below which no excess mortality is assumed to occur; (2) we used unmodified effect estimates (i.e., assuming Σ*F_j_*×*t_j_* = 1 in Equation 4 in the main text) rather than our modified effect estimates; and (3) we used both our primary all-cause mortality endpoint effect estimate (i.e., *β* = 0.0070 per µg/m^3^)^17^ and a lower effect estimate that has been used in other recent studies (i.e., *β* = 0.0058 per µg/m^3^)^5^ because we are uncertain as to which effect estimate would yield similar mortality predictions as the IER approach with RRs from Apte et al. (2015).^37^

Results from the two additional Scenario 1 case studies are shown in Table S4 below the GBD IER case studies. The median total mortality estimates using the generic E-R model (Equation 1 in the main text) with *β* = 0.0070 per µg/m^3^ and *β* = 0.0058 per µg/m^3^ were ~42,600 and ~36,000 deaths annually, respectively. Results from the IER model application were approximately in between these two estimates, which suggests that the application of the IER model yields mortality estimates that are approximately equivalent to applying the E-R model form with the assumption of an unmodified total all-cause mortality excess RR of ~6 to ~7% per 10 µg/m^3^ with a threshold of 5.8 µg/m^3^. Importantly, the similarities in both model results using the same or equivalent inputs also suggests that the vast majority of the difference in mortality estimates between Scenario 3 and Scenarios 1 and 2 is driven by the assumption of a 5.8 µg/m^3^ threshold concentration in Scenario 3 compared to a zero threshold concentration in Scenarios 1 and 2. This is a critical discrepancy that the research community must address if analyses like this and others are to be relied upon for informing high-level policy decisions. Our original assumption of a no threshold concentration is consistent with a number of studies that have demonstrated or suggested that there is no evidence of a population threshold in the relationship between long-term exposure to ambient PM_2.5_ and mortality.^8,45–47^ However, the majority of applications of the IER model have assumed either a threshold concentration of 5.8 µg/m^3^ (e.g., ^36,42^) or a distribution between 2.4 and 5.8 µg/m^3^ (e.g., ^39^). Since a large portion of the mortality burden estimated using these types of risk assessment functions is attributable to PM_2.5_ concentrations at the lower end of the curve, these two modeling assumptions can yield vastly different mortality estimates.

# References

1 Di Q, Wang Y, Zanobetti A, Wang Y, Koutrakis P, Choirat C *et al.* Air Pollution and Mortality in the Medicare Population. *New England Journal of Medicine* 2017; **376**: 2513–2522.

2 Dockery DW, Pope CA 3rd, Xu X, Spengler JD, Ware JH, Fay ME *et al.* An association between air pollution and mortality in six U.S. cities. *N Engl J Med* 1993; **329**: 1753–1759.

3 Jerrett M, Burnett RT, Ma R, Pope CA, Krewski D, Newbold KB *et al.* Spatial Analysis of Air Pollution and Mortality in Los Angeles: *Epidemiology* 2005; **16**: 727–736.

4 Krewski D, Jerrett M, Rt B, R M, E H, Y S *et al.* Extended follow-up and spatial analysis of the American Cancer Society study linking particulate air pollution and mortality. *Res Rep Health Eff Inst* 2009; : 5–114; discussion 115-36.

5 Laden F, Schwartz J, Speizer FE, Dockery DW. Reduction in Fine Particulate Air Pollution and Mortality: Extended Follow-up of the Harvard Six Cities Study. *Am J Respir Crit Care Med* 2006; **173**: 667–672.

6 Pope CA, Thun MJ, Namboodiri MM, Dockery DW, Evans JS, Speizer FE *et al.* Particulate Air Pollution as a Predictor of Mortality in a Prospective Study of U.S. Adults. *Am J Respir Crit Care Med* 1995; **151**: 669–674.

7 Pope CA, Burnett RT, Thun MJ, Calle EE, Krewski D, Ito K *et al.* Lung cancer, cardiopulmonary mortality, and long-term exposure to fine particulate air pollution. *JAMA* 2002; **287**: 1132–1141.

8 Roman HA, Walker KD, Walsh TL, Conner L, Richmond HM, Hubbell BJ *et al.* Expert Judgment Assessment of the Mortality Impact of Changes in Ambient Fine Particulate Matter in the U.S. *Environmental Science & Technology* 2008; **42**: 2268–2274.

9 US EPA. The Benefits and Costs of the Clean Air Act from 1990 to 2020. U.S. Environmental Protection Agency Office of Air and Radiation: Washington, DC, 2011http://www.epa.gov/cleanairactbenefits/feb11/fullreport_rev_a.pdf.

10 Eftim SE, Samet JM, Janes H, McDermott A, Dominici F. Fine particulate matter and mortality: a comparison of the six cities and American Cancer Society cohorts with a medicare cohort. *Epidemiology* 2008; **19**: 209–216.

11 Hodas N, Meng Q, Lunden MM, Rich DQ, Özkaynak H, Baxter LK *et al.* Variability in the fraction of ambient fine particulate matter found indoors and observed heterogeneity in health effect estimates. *Journal of Exposure Science and Environmental Epidemiology* 2012; **22**: 448–454.

12 Puett RC, Schwartz J, Hart JE, Yanosky JD, Speizer FE, Suh H *et al.* Chronic particulate exposure, mortality, and coronary heart disease in the nurses’ health study. *Am J Epidemiol* 2008; **168**: 1161–1168.

13 Zeger SL, Dominici F, McDermott A, Samet JM. Mortality in the Medicare population and chronic exposure to fine particulate air pollution in urban centers (2000-2005). *Environ Health Perspect* 2008; **116**: 1614–1619.

14 Chen C, Zhao B, Weschler CJ. Indoor exposure to “outdoor PM10”. *Epidemiology* 2012; **23**: 870–878.

15 Ji W, Zhao B. Estimating mortality derived from indoor exposure to particles of outdoor origin. *PLOS ONE* 2015; **10**: e0124238.

16 Bell ML, Ebisu K, Peng RD, Dominici F. Adverse Health Effects of Particulate Air Pollution: Modification by Air Conditioning. *Epidemiology* 2009; **20**: 682–686.

17 Fann N, Gilmore EA, Walker K. Characterizing the Long-Term PM _2.5_ Concentration-Response Function: Comparing the Strengths and Weaknesses of Research Synthesis Approaches: Characterizing Long-Term PM _2.5_ Concentration-Response Function. *Risk Analysis* 2016; **36**: 1693–1707.

18 Hoek G, Krishnan RM, Beelen R, Peters A, Ostro B, Brunekreef B *et al.* Long-term air pollution exposure and cardio- respiratory mortality: a review. *Environmental Health* 2013; **12**. doi:10.1186/1476-069X-12-43.

19 Rackes A, Ben-David T, Waring MS. Outcome-based ventilation: A framework for assessing performance, health, and energy impacts to inform office building ventilation decisions. *Indoor Air* 2018. doi:10.1111/ina.12466.

20 Klepeis NE, Nelson WC, Ott WR, Robinson JP, Tsang AM, Switzer P *et al.* The National Human Activity Pattern Survey (NHAPS): a resource for assessing exposure to environmental pollutants. *J Expo Anal Environ Epidemiol* 2001; **11**: 231–252.

21 Chen C, Zhao B. Review of relationship between indoor and outdoor particles: I/O ratio, infiltration factor and penetration factor. *Atmos Environ* 2011; **45**: 275–288.

22 Ben-David T, Rackes A, Waring MS. Alternative ventilation strategies in U.S. offices: Saving energy while enhancing work performance, reducing absenteeism, and considering outdoor pollutant exposure tradeoffs. *Building and Environment* 2017; **116**: 140–157.

23 Hänninen OO, Palonen J, Tuomisto JT, Yli-Tuomi T, Seppanen O, Jantunen MJ. Reduction potential of urban PM2.5 mortality risk using modern ventilation systems in buildings. *Indoor Air* 2005; **15**: 246–256.

24 Fromme H, Diemer J, Dietrich S, Cyrys J, Heinrich J, Lang W *et al.* Chemical and morphological properties of particulate matter (PM10, PM2.5) in school classrooms and outdoor air. *Atmospheric Environment* 2008; **42**: 6597–6605.

25 Rivas I, Viana M, Moreno T, Bouso L, Pandolfi M, Alvarez-Pedrerol M *et al.* Outdoor infiltration and indoor contribution of UFP and BC, OC, secondary inorganic ions and metals in PM 2.5 in schools. *Atmospheric Environment* 2015; **106**: 129–138.

26 Hudda N, Fruin SA. Models for Predicting the Ratio of Particulate Pollutant Concentrations Inside Vehicles to Roadways. *Environmental Science & Technology* 2013; **47**: 11048–11055.

27 Wu X, Apte MG, Bennett DH. Indoor Particle Levels in Small- and Medium-Sized Commercial Buildings in California. *Environmental Science & Technology* 2012; **46**: 12355–12363.

28 Karner AA, Eisinger DS, Niemeier DA. Near-Roadway Air Quality: Synthesizing the Findings from Real-World Data. *Environmental Science & Technology* 2010; **44**: 5334–5344.

29 Weisel CP, Zhang J, Turpin BJ, Morandi MT, Colome S, Stock TH *et al.* Relationship of Indoor, Outdoor and Personal Air (RIOPA) study: study design, methods and quality assurance/control results. *Journal of Exposure Analysis and Environmental Epidemiology* 2004; **15**: 123–137.

30 Cohen MA, Adar SD, Allen RW, Avol E, Curl CL, Gould T *et al.* Approach to Estimating Participant Pollutant Exposures in the Multi-Ethnic Study of Atherosclerosis and Air Pollution (MESA Air). *Environ Sci Technol* 2009; **43**: 4687–4693.

31 Kaufman JD, Adar SD, Allen RW, Barr RG, Budoff MJ, Burke GL *et al.* Prospective Study of Particulate Air Pollution Exposures, Subclinical Atherosclerosis, and Clinical Cardiovascular Disease: The Multi-Ethnic Study of Atherosclerosis and Air Pollution (MESA Air). *American Journal of Epidemiology* 2012; **176**: 825–837.

32 Meng QY, Turpin BJ, Korn L, Weisel CP, Morandi M, Colome S *et al.* Influence of ambient (outdoor) sources on residential indoor and personal PM2.5 concentrations: Analyses of RIOPA data. *J Expo Anal Environ Epidemiol* 2005; **15**: 17–28.

33 Meng QY, Turpin BJ, Polidori A, Lee JH, Weisel C, Morandi M *et al.* PM2.5 of ambient origin: estimates and exposure errors relevant to PM epidemiology. *Environmental Science & Technology* 2005; **39**: 5105–5112.

34 Allen RW, Adar SD, Avol E, Cohen M, Curl CL, Larson T *et al.* Modeling the residential infiltration of outdoor PM2.5 in the Multi-Ethnic Study of Atherosclerosis and Air Pollution (MESA Air). *Environmental Health Perspectives* 2012; **120**: 824–830.

35 Fann N, Kim S-Y, Olives C, Sheppard L. Estimated Changes in Life Expectancy and Adult Mortality Resulting from Declining PM2.5 Exposures in the Contiguous United States: 1980–2010. *Environmental Health Perspectives* 2017; **125**. doi:10.1289/EHP507.

36 Apte JS, Marshall JD, Cohen AJ, Brauer M. Addressing Global Mortality from Ambient PM_2.5_. *Environmental Science & Technology* 2015; **49**: 8057–8066.

37 Lelieveld J, Evans JS, Fnais M, Giannadaki D, Pozzer A. The contribution of outdoor air pollution sources to premature mortality on a global scale. *Nature* 2015; **525**: 367–371.

38 Rohde RA, Muller RA. Air Pollution in China: Mapping of Concentrations and Sources. *PLOS ONE* 2015; **10**: e0135749.

39 Cohen AJ, Brauer M, Burnett R, Anderson HR, Frostad J, Estep K *et al.* Estimates and 25-year trends of the global burden of disease attributable to ambient air pollution: an analysis of data from the Global Burden of Diseases Study 2015. *The Lancet* 2017; **389**: 1907–1918.

40 Chowdhury S, Dey S, Smith KR. Ambient PM2.5 exposure and expected premature mortality to 2100 in India under climate change scenarios. *Nature Communications* 2018; **9**. doi:10.1038/s41467-017-02755-y.

41 Burnett RT, Pope CA III, Ezzati M, Olives C, Lim SS, Mehta S *et al.* An Integrated Risk Function for Estimating the Global Burden of Disease Attributable to Ambient Fine Particulate Matter Exposure. *Environmental Health Perspectives* 2014. doi:10.1289/ehp.1307049.

42 Wang J, Xing J, Mathur R, Pleim JE, Wang S, Hogrefe C *et al.* Historical Trends in PM2.5-Related Premature Mortality during 1990–2010 across the Northern Hemisphere. *Environmental Health Perspectives* 2016; **125**. doi:10.1289/EHP298.

43 Burnett R, Chen H, Szyszkowicz M, Fann N, Hubbell B, Pope CA *et al.* Global estimates of mortality associated with long-term exposure to outdoor fine particulate matter. *PNAS* 2018; : 201803222.

44 CDC. National Center for Health Statistics WONDER Online Database: Compressed Mortality File 1999-2016 Series 20, No. 2V. CDC WONDER. 2017.https://wonder.cdc.gov/cmf-icd10.htm (accessed 22 May2018).

45 Crouse DL, Peters PA, van Donkelaar A, Goldberg MS, Villeneuve PJ, Brion O *et al.* Risk of Nonaccidental and Cardiovascular Mortality in Relation to Long-term Exposure to Low Concentrations of Fine Particulate Matter: A Canadian National-Level Cohort Study. *Environmental Health Perspectives* 2012; **120**: 708–714.

46 Schwartz J, Coull B, Laden F, Ryan L. The Effect of Dose and Timing of Dose on the Association between Airborne Particles and Survival. *Environmental Health Perspectives* 2007; **116**: 64–69.

47 Pinault L, Tjepkema M, Crouse DL, Weichenthal S, van Donkelaar A, Martin RV *et al.* Risk estimates of mortality attributed to low concentrations of ambient fine particulate matter in the Canadian community health survey cohort. *Environmental Health* 2016; **15**. doi:10.1186/s12940-016-0111-6.

# Figures

Figure S1. Beta distributions of PM_2.5_ infiltration factors used in Scenario 1: a) two residential distributions (RIOPA and MESA) and b) other indoor environments and vehicles (which were also used in Scenario 2). Distribution shape factors are described in the SI text.

# Tables

**Table S1. Summary of stair-step distribution characteristics for the amount of time spent in various microenvironments based on Figure 3 in Klepeis et al. (2001), used in all model scenarios**

|  | Residences | | Vehicles | | Outdoors | | Other indoor locations | |
| --- | --- | --- | --- | --- | --- | --- | --- | --- |
|  | Reported | Modeled | Reported | Modeled | Reported | Modeled | Reported | Modeled |
| Average(min) | 990 | 978 | 79 | 59 | 109 | 98 | 262 | 305 |
|  | | | | | | | | |
| Bin # | Range (min) | Prob. | Range (min) | Prob. | Range (min) | Prob. | Range (min) | Prob. |
| 1 | 0-50 | 0.0085 | 0-60 | 0.6475 | 0-60 | 0.5564 | Back-calculated | Back-calculated |
| 2 | 50-100 | 0.0028 | 60-120 | 0.0965 | 60-120 | 0.2700 |  |  |
| 3 | 100-150 | 0.0028 | 120-180 | 0.0727 | 120-180 | 0.1080 |  |  |
| 4 | 150-200 | 0.0038 | 180-240 | 0.0441 | 180-240 | 0.0240 |  |  |
| 5 | 200-250 | 0.0038 | 240-300 | 0.0330 | 240-300 | 0.0120 |  |  |
| 6 | 250-300 | 0.0038 | 300-360 | 0.0240 | 300-360 | 0.0090 |  |  |
| 7 | 300-350 | 0.0038 | 360-420 | 0.0240 | 360-420 | 0.0054 |  |  |
| 8 | 350-400 | 0.0058 | 420-480 | 0.0120 | 420-480 | 0.0054 |  |  |
| 9 | 400-450 | 0.0068 | 480-540 | 0.0120 | 480-540 | 0.0025 |  |  |
| 10 | 450-500 | 0.0125 | 540-600 | 0.0120 | 540-600 | 0.0025 |  |  |
| 11 | 500-550 | 0.0125 | 600-660 | 0.0090 | 600-660 | 0.0025 |  |  |
| 12 | 550-600 | 0.0200 | 660-720 | 0.0048 | 660-720 | 0.0025 |  |  |
| 13 | 600-650 | 0.0300 | 720-780 | 0.0030 | 720-780 | 0.0000 |  |  |
| 14 | 650-700 | 0.0400 | 780-840 | 0.0018 | 780-840 | 0.0000 |  |  |
| 15 | 700-750 | 0.0500 | 840-900 | 0.0018 | 840-900 | 0.0000 |  |  |
| 16 | 750-800 | 0.0655 | 900-960 | 0.0018 | 900-960 | 0.0000 |  |  |
| 17 | 800-850 | 0.0655 | 960-1020 | 0.0000 | 960-1020 | 0.0000 |  |  |
| 18 | 850-900 | 0.0675 | 1020-1080 | 0.0000 | 1020-1080 | 0.0000 |  |  |
| 19 | 900-950 | 0.0615 | 1080-1140 | 0.0000 | 1080-1140 | 0.0000 |  |  |
| 20 | 950-1000 | 0.0500 | 1140-1200 | 0.0000 | 1140-1200 | 0.0000 |  |  |
| 21 | 1000-1050 | 0.0450 | 1200-1260 | 0.0000 | 1200-1260 | 0.0000 |  |  |
| 22 | 1050-1100 | 0.0496 | 1260-1320 | 0.0000 | 1260-1320 | 0.0000 |  |  |
| 23 | 1100-1150 | 0.0450 | 1320-1380 | 0.0000 | 1320-1380 | 0.0000 |  |  |
| 24 | 1150-1200 | 0.0475 | 1380-1440 | 0.0000 | 1380-1440 | 0.0000 |  |  |
| 25 | 1200-1250 | 0.0447 |  | |  | |  |  |
| 26 | 1250-1300 | 0.0466 |  |  |  |  |  |  |
| 27 | 1300-1350 | 0.0418 |  |  |  |  |  |  |
| 28 | 1350-1400 | 0.0428 |  |  |  |  |  |  |
| 29 | 1400-1440 | 0.1205 |  |  |  |  |  |  |

Table S2. Summary of distributions of residential indoor and outdoor PM_2.5_ concentrations, PM_2.5_ infiltration factors, and indoor PM_2.5_ concentrations attributable to outdoor sources in the RIOPA and MESA Air studies that were used to fit distributions to the indoor PM_2.5_ concentrations that result from indoor sources in Scenario 1

| **Study** | **Reference** | **Model Parameter** | **Distribution**  **Type** | **Mean ± SD**  **(µg/m^3^)^*^** |
| --- | --- | --- | --- | --- |
| RIOPA | Meng et al. (2005)^32^ | Indoor PM_2.5_ concentration | Lognormal | GM: 15.6  GSD: 1.7 |
|  |  | Outdoor PM_2.5_ concentration | Lognormal | GM: 14.3  GSD: 1.9 |
|  | Meng et al. (2005)^33^ | PM_2.5_ infiltration factor | Beta | 0.54 ± 0.16 |
|  | *Calculated* | Indoor PM_2.5_ concentration attributable to indoor sources | n/a | 8.7 ± 11.8 |
| MESA  Air | Allen et al. (2012)^34^ | Indoor PM_2.5_ concentration (cold season) | n/a | 10.4 ± 7.0 |
|  |  | Indoor PM_2.5_ concentration (warm season) | n/a | 12.8 ± 5.6 |
|  |  | Outdoor PM_2.5_ concentration (cold season) | n/a | 13.5 ± 5.8 |
|  |  | Outdoor PM_2.5_ concentration (warm season) | n/a | 15.8 ± 3.9 |
|  |  | Indoor PM_2.5_ concentration attributable to indoor sources (cold season) | n/a | 2.8 ± 4.8 |
|  |  | Indoor PM_2.5_ concentration attributable to indoor sources (warm season) | n/a | 2.7 ± 4.0 |
|  |  | PM_2.5_ infiltration factor | Beta | 0.62 ± 0.21 |
|  | *Calculated* | Indoor PM_2.5_ concentration attributable to indoor sources (combined seasons) | Lognormal | GM: 1.5  GSD: 3.1 |

*For lognormal distributions, GM = geometric mean and GSD = geometric standard deviation.

n/a = values reported in the cited references but not used directly in the model application.

Table S3. Summary of the IER model input parameters used herein for adults 35 years and older. Data taken from the CDC WONDER system.^43^

| **Cause of Mortality** | **Age ranges** | **Population in 2012** | **Average disease incidence (y_0_)** |
| --- | --- | --- | --- |
| COPD | + 35 | 166,516,716 | 0.000840 |
| Lung Cancer | + 35 | 166,516,716 | 0.000948 |
| IHD | 35 – 39 | 19,488,199 | 0.000082 |
|  | 40 – 44 | 21,028,221 | 0.000171 |
|  | 45 – 49 | 21,689,479 | 0.000354 |
|  | 50 – 54 | 22,579,259 | 0.000631 |
|  | 55 – 59 | 20,772,517 | 0.000996 |
|  | 60 – 64 | 17,813,685 | 0.001517 |
|  | 65 – 69 | 13,977,353 | 0.002159 |
|  | 70 – 74 | 10,008,039 | 0.003260 |
|  | 75 – 79 | 7,489,583 | 0.005340 |
|  | + 80 | 11,670,381 | 0.016506 |
| Stroke | 35 – 39 | 19,488,199 | 0.000029 |
|  | 40 – 44 | 21,028,221 | 0.000053 |
|  | 45 – 49 | 21,689,479 | 0.000097 |
|  | 50 – 54 | 22,579,259 | 0.000146 |
|  | 55 – 59 | 20,772,517 | 0.000219 |
|  | 60 – 64 | 17,813,685 | 0.000334 |
|  | 65 – 69 | 13,977,353 | 0.000523 |
|  | 70 – 74 | 10,008,039 | 0.000961 |
|  | 75 – 79 | 7,489,583 | 0.001789 |
|  | + 80 | 11,670,381 | 0.005845 |

Table S4. Mean, standard deviation (SD), and interquartile range (IQR: 25^th^ to 75^th^ percentiles) of the estimated contributions to total PM_2.5_ exposures resulting from IER and E-R models with unmodified effect estimates and a threshold concentration of 5.8 μg/m^3^ for the U.S. population 35 years and older in 2012.

| **Applied Model** | **Outdoor or Indoor Sources** | **Microenvironment** | **Mean fraction of total PM_2.5_ exposure ±** **SD** | **IQR of fraction of total PM_2.5_ exposure** | **Mean estimate of annual deaths attributed to total PM_2.5_ exposure ±** **SD** | **IQR of estimate of annual deaths attributed to total PM_2.5_ exposure** |
| --- | --- | --- | --- | --- | --- | --- |
| **Unmodified IER model** | Due to PM_2.5_ of outdoor origin | Residences | 41.9% **±** 24.1% | 21.9% - 58.7% | 16,089 **±** 9,244 | 8,410 - 22,545 |
|  |  | Other indoor locations | 10.8% **±** 12.8% | 0.0% - 16.8% | 4,144 **±** 4,928 | 0 - 6,460 |
|  |  | Vehicles | 2.4% **±** 4.0% | 0.3% - 2.7% | 916 **±** 1,524 | 120 - 1,023 |
|  |  | Outdoor | 7.4% **±** 11.3% | 1.7% - 6.9% | 2,840 **±** 4,355 | 667 - 2,649 |
|  | Total outdoor contribution | | 62.5% **±** 25.4% | | 23,989 **±** 9,756 | 9,196 - 32,677 |
|  | Due to PM_2.5_ of indoor origin | Residences | 28.2% **±** 26.5% | 5.2% - 47.9% | 10,838 **±** 10,159 | 2,011 - 18,397 |
|  |  | Other indoor locations | 9.3% **±** 13.0% | 0.0% - 13.0% | 3,554 **±** 4,991 | 0 - 5,002 |
|  | Total indoor contribution | | 37.5% **±** 25.4% | | 14,393 **±** 9,756 | 2,011 - 23,398 |
|  | Total contribution | | 100% | | 38,382^*^ | 11,208 - 56,075 |
| **Unmodified E-R model (*β* = 0.0070 per µg/m^3^)** | Due to PM_2.5_ of outdoor origin | Residences | 42.0% **±** 24.0% | 22.3% - 58.8% | 17,903 **±** 10,231 | 9,526 - 25,049 |
|  |  | Other indoor locations | 10.9% **±** 13.0% | 0.0% - 16.8% | 4,648 **±** 5,534 | 0 - 7,144 |
|  |  | Vehicles | 2.4% **±** 4.0% | 0.3% - 2.7% | 1,029 **±** 1,710 | 130 - 1,164 |
|  |  | Outdoor | 7.2% **±** 11.2% | 1.7% - 6.4% | 3,052 **±** 4,762 | 745 - 2,738 |
|  | Total outdoor contribution | | 62.5% **±** 25.3% | | 26,632 **±** 10,766 | 10,401 - 36,096 |
|  | Due to PM_2.5_ of indoor origin | Residences | 28.3% **±** 26.5% | 5.1% - 48.2% | 12,054 **±** 11,290 | 2,193 - 20,546 |
|  |  | Other indoor locations | 9.2% **±** 13.0% | 0.0% - 12.7% | 3,937 **±** 5,532 | 0 - 5,395 |
|  | Total indoor contribution | | 37.5% **±** 25.3% | | 15,991 **±** 10,766 | 2,193 - 25,940 |
|  | Total contribution | | 100% | | 42,623^*^ | 12,594 - 62,036 |
| **Unmodified E-R model (*β* = 0.0058 per µg/m^3^)** | Due to PM_2.5_ of outdoor origin | Residences | 41.9% **±** 23.8% | 22.6% - 58.6% | 15,097 **±** 8,590 | 8,155 - 21,118 |
|  |  | Other indoor locations | 10.9% **±** 12.9% | 0.1% - 17.0% | 3,921 **±** 4,644 | 22 - 6,131 |
|  |  | Vehicles | 2.3% **±** 3.7% | 0.3% - 2.7% | 844 **±** 1,346 | 126 - 979 |
|  |  | Outdoor | 7.5% **±** 11.5% | 1.8% - 7.0% | 2,688 **±** 4,128 | 640 - 2,535 |
|  | Total outdoor contribution | | 62.6% **±** 25.2% | | 22,550 **±** 9,079 | 8,942 - 30,763 |
|  | Due to PM_2.5_ of indoor origin | Residences | 28.1% **±** 26.3% | 5.2% - 48.2% | 10,108 **±** 9,479 | 1,860 - 17,359 |
|  |  | Other indoor locations | 9.4% **±** 13.0% | 0.0% - 13.2% | 3,371 **±** 4,669 | 13 - 4,755 |
|  | Total indoor contribution | | 37.4% **±** 25.2% | | 13,478 **±** 9,079 | 1,873 - 22,114 |
|  | Total contribution | | 100% | | 36,028^*^ | 10,815 - 52,877 |

^*^ The estimates of total contributions are based on the median values.
